# Recurrent Hemothorax and Hemoperitoneum in Endometriosis: A Case Report

**DOI:** 10.7759/cureus.89503

**Published:** 2025-08-06

**Authors:** Sarah S Park, Justin Peterson

**Affiliations:** 1 Department of Medicine, David Geffen School of Medicine, University of California, Los Angeles, Los Angeles, USA

**Keywords:** catamenial hemothorax, endometriosis complications, extra-pelvic endometriosis, exudative pleural effusion, hemoperitoneum, internal bleeding, thoracic endometriosis

## Abstract

Extra-pelvic endometriosis, defined as the presence of endometrial tissue or stroma outside of the pelvic cavity, is a rare cause of hemothorax and hemoperitoneum. Here, we present a case of a 34-year-old woman with a history of endometriosis who experienced recurrent, cyclical episodes of hemorrhagic pleural effusions and ascites. Despite multiple surgeries to address the ectopic endometrial implants, her symptoms persisted for years without definitive resolution, illustrating the challenges of treatment even after the diagnosis has been established.

This case highlights the rare manifestations of extra-pelvic endometriosis and the long-term complications they may pose. Early recognition and intervention are crucial to prevent chronic sequelae and reduce morbidity.

## Introduction

Endometriosis is a common gynecologic disorder, present in approximately 10% of reproductive-age women [1-3]. It is characterized by ectopic endometrial tissue growth in various areas outside of the uterus. These implants respond to hormonal fluctuations, undergoing cyclical proliferation and shedding, which can result in bleeding, chronic inflammation, and adhesions. While the pelvis is the most common site, most commonly the fallopian tubes, ovaries, uterosacral ligaments, and rectouterine pouch, ectopic endometrial tissue can also be found in extra-pelvic sites, such as the gastrointestinal tract, urinary tract, peritoneum, lungs, diaphragm, and nerves, accounting for about 10-20% of cases [4-7].

The clinical presentation of endometriosis can vary widely based on the site of implantation. Classic symptoms include dysmenorrhea, dyspareunia, and chronic pelvic pain associated with menses; however, patients may also be asymptomatic or present later on with infertility. A minority experience atypical manifestations such as gastrointestinal symptoms (bloating, dyschezia), urinary symptoms (dysuria, hematuria), thoracic symptoms (dyspnea, pleuritic chest pain), and neurologic symptoms (sciatica) [1-3,5,7].

Thoracic endometriosis syndrome (TES) is a less commonly seen subtype of extra-pelvic endometriosis, caused by the presence of endometrial tissue within the thoracic cavity and/or pulmonary parenchyma. It most often presents as catamenial pneumothorax, followed by hemothorax or catamenial pleural effusion. Hemoptysis and pulmonary nodules are uncommon manifestations of this syndrome [8]. Endometriosis-related ascites is an even rarer complication of extra-pelvic endometriosis. Associated inflammation, as well as potential lymphatic obstruction, may impair drainage, leading to large-volume ascites [9,10]. The prevalence of both TES and ascites due to endometriosis is rarely seen in the same patient [11]. The diagnosis of ascites and/or pleural effusions caused by endometriosis is often a diagnostic challenge given its rare prevalence, overlap with other diagnoses, and multiple challenges associated with achieving pathologic confirmation.

## Case presentation

A 34-year-old woman with a history of endometriosis presented to the emergency department with progressive right-sided chest pain and dyspnea that began two days prior to presentation. The chest pain was described as severe (10/10 at rest), radiating to her neck and back, and exacerbated by deep inspiration, movement, and positional changes. Non-steroidal anti-inflammatory drugs (NSAIDs) did not provide relief. She had also recently tested positive for severe acute respiratory syndrome coronavirus 2 (SARS-CoV-2).

On review of systems, she denied fever, chills, sweats, cough, sputum production, or other signs of a respiratory infection. She denied any palpitations, headache, dizziness, or syncope. Her only medical history was severe endometriosis, diagnosed via laparoscopy in 2018. At that time, the patient had a resection of an endometrioma and peritoneal endometrial implants, but soon thereafter, she experienced "bleeding from her belly button" and underwent a second surgical excision for endometrial implants around her umbilicus. Following these surgeries, she experienced two episodes of hemorrhagic ascites in 2021 and 2022, requiring diagnostic and therapeutic paracenteses.

**Figure 1:**
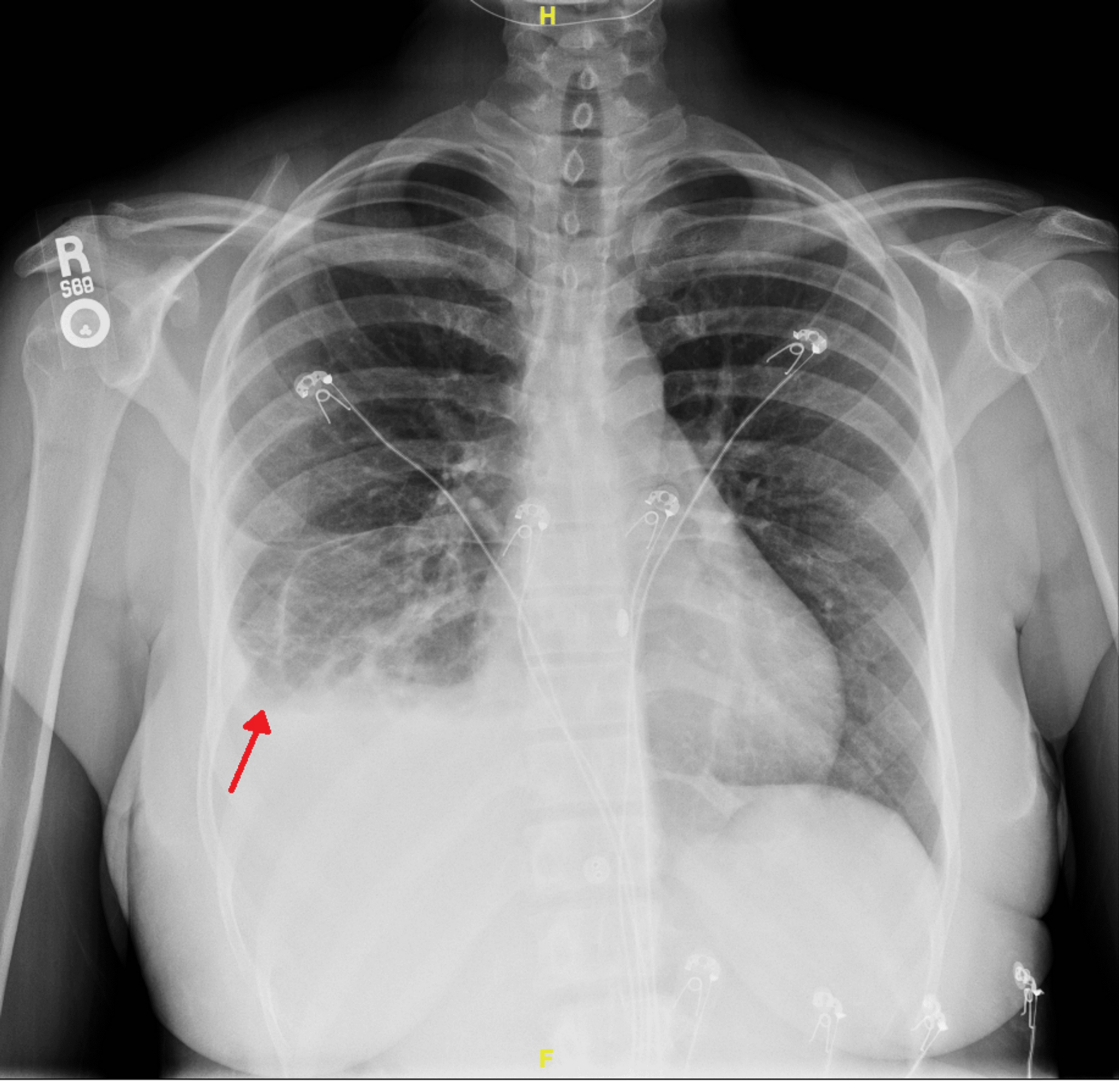
Chest radiography demonstrating a moderate right pleural effusion with associated compressive atelectasis and consolidation (identified by the red arrow).

In the emergency department, she was mildly tachycardic but was otherwise afebrile, normotensive, and saturating well on room air. The physical exam was only notable for diminished breath sounds at the right base, without any wheezing, crackles, or respiratory distress. Laboratory tests on admission are shown in Table 1. Electrocardiogram revealed a normal sinus rhythm. A chest X-ray showed a moderate right pleural effusion with consolidation and atelectasis (Figure 1). Despite the lack of infectious symptoms and unusual presentation for viral pneumonia, she was admitted for presumed COVID-19 pneumonia and empirically started on intravenous antibiotics for possible bacterial superinfection.

**Table 1:** Laboratory values. SARS-CoV-2 RNA PCR: severe acute respiratory syndrome coronavirus 2 ribonucleic acid polymerase chain reaction.

Laboratory test	Result	Reference range
White blood cell count	6.6 ×10^3^/uL	4.16 - 9.95 x10^3^/uL
Hemoglobin	9.4 g/dL	11.6 - 15.2 g/dl
Platelets	312 ×10^3^/uL	143 - 398 x10^3^/uL
Sodium	140 mmol/L	135 - 146 mmol/L
Potassium	3.5 mmol/L	3.6 - 5.3 mmol/L
Chloride	108 mmol/L	96 - 106 mmol/L
Bicarbonate	26 mmol/L	20 - 30 mmol/L
Blood urea nitrogen (BUN)	14 mg/dL	7 - 22 mg/dL
Creatinine	0.64 mg/dL	0.6 - 1.3 mg/dL
Albumin	3.6 g/dL	3.9 - 5.0 g/dL
Total protein	8.6 g/dL	6.1 - 8.2 g/dL
Procalcitonin	<0.05 ug/L	<0.10 ug/L
International normalized ratio (INR)	1.22	0.80 - 1.19
Prothrombin time	15.8 seconds	11.2 - 15.5 seconds
SARS-CoV-2 RNA PCR	Positive	Negative

The following day, a diagnostic and therapeutic thoracentesis yielded 1000 cc of grossly bloody fluid. Analysis of the pleural fluid (Table 2) was consistent with an exudative effusion per Light's criteria. Further history revealed that the date of her last menstrual period was two days prior to admission, the same day as when her symptoms began. Given the temporal onset and resolution of symptoms associated with her menstrual cycle, the pleural effusion was attributed to thoracic endometriosis, and she was subsequently discharged.

**Table 2:** Pleural fluid analysis.

Laboratory test	Result
Total volume	1000 mL
Color	Red
pH	7.38
White blood cell count	1150/cu mm
Segmented cells	27%
Lymphocytes	21%
Monocytes	52%
Mesothelial cells	Present
Total protein	4.9 g/dL
Lactate dehydrogenase	668 U/L

Months later, she presented again with similar right-sided chest pain and dyspnea. She reported trying oral contraceptives (OCPs) for the first time following discharge, but they were stopped due to an allergic reaction, described as “eye and mouth swelling with itchiness and redness.” Given her reaction to hormonal therapy, she was instead placed on a non-hormonal medication, which was ineffective at controlling her menses. A subsequent chest X-ray revealed another right-sided pleural effusion, and repeat thoracentesis was performed to drain 750 cc of grossly bloody fluid. She was subsequently discharged.

However, she returned two weeks later with unresolved chest pain and dyspnea from prior. She had developed another right-sided pleural effusion. She was no longer menstruating, yet her pleuritic chest pain was still present. To address the recurrent pleural effusions, a pleuroscopy with pleural biopsy was performed. Intraoperatively, 200 cc of serosanguinous fluid was aspirated, and numerous visible adhesions were lysed. Reports showed no evidence of bacterial, fungal, mycobacterial, or malignant etiologies, nor any evidence of endometriosis. The final histopathology report revealed “fibrinous pleuritis with reactive mesothelial hyperplasia.”

She later underwent an exploratory laparotomy to address the recurrent ascites and associated symptoms, with a plan for potential total abdominal hysterectomy with bilateral salpingo-oophorectomy for definitive management. While no distinct endometriotic surfaces were identified, a 2 x 2 cm intramural mass near the ileocecal valve was palpated, indicating a possible endometriotic mass. Extensive pelvic adhesions were also noted. The uterus, adnexa (including the ovaries), and sigmoid colon were densely adherent, appearing to be “frozen to one conglomerate,” likely reflecting sequelae of her advanced endometriosis. Multiple attempts were made to mobilize the organs, but all were unsuccessful. Ultimately, it was concluded that full resection would require pelvic exenteration with sigmoid resection and reanastomosis.

## Discussion

This case highlights the variable manifestations of extra-pelvic endometriosis, a rare and often underrecognized cause of recurrent internal bleeding in reproductive-age women. Symptoms can easily be misattributed to non-gynecologic causes, as shown here with our patient’s hemorrhagic pleural effusion that was initially attributed to viral pneumonia despite a lack of infectious symptoms. However, it is crucial to maintain a high index of suspicion for recurrent, unexplained hemorrhagic effusions in premenopausal women, especially when episodes coincide with menses.

Thoracic endometriosis can manifest as a pneumothorax, hemothorax, or an exudative pleural effusion. Symptoms typically occur 24 hours before or within 72 hours after the onset of menses, though occurrences outside of this time frame are possible. The vast majority of catamenial effusions occur in the right hemithorax for a variety of proposed mechanisms, including clockwise flow of peritoneal fluid, pressure gradients from the liver, diaphragmatic defects, and other anatomic differences between the right and left hemidiaphragms [12-15].

Like an exudative pleural effusion, endometriosis-related ascites is typically bloody and protein-rich, with a low serum-ascites albumin gradient (SAAG), reflecting its inflammatory, non-portal hypertensive origin [16]. Moreover, markedly elevated CA-125 levels can be seen in endometrial ascites without underlying malignancy, further clouding the diagnostic picture [9,10,16,17]. The distinction of this entity from malignancy-associated ascites, particularly ovarian malignancy, is paramount, as there can be substantial clinical overlap.

Even with a high index of suspicion, diagnosing extra-pelvic endometriosis can be challenging. While imaging and fluid analyses can support the diagnosis, a surgical biopsy is required for definitive confirmation. However, tissue examination may be inconclusive given the cyclical nature of lesions, sampling error, and anatomically inaccessible areas for biopsy [18]. Iatrogenic spread of endometriosis during laparoscopic biopsy is also common, as postsurgical scars are thought to be prime locations for implantation of endometrial cells [5]. In our patient, the development of umbilical endometrial implants following laparoscopic biopsy suggests such a possibility.

Given the diagnostic challenges, clinical suspicion alone is often sufficient to initiate treatment. First-line treatment involves NSAIDs and hormonal therapy. Medical therapy may alleviate bleeding and pain, but it is not always well-tolerated and will not improve fertility. As a result, surgical management is often recommended in refractory cases or for those desiring fertility, but recurrence after excision is common, and even a hysterectomy does not guarantee complete resolution [19-22].

Persistent endometriotic lesions that are refractory to medical treatment can lead to further complications. As blood within internal cavities is highly inflammatory, recurrent episodes of internal bleeding can lead to repeated cycles of inflammation, injury, and repair. In the thoracic cavity, the presence of blood can irritate the pleural lining and lead to fibrin deposition, resulting in fibrinous pleuritis as well as reactive mesothelial cell hyperplasia, consistent with the intra-thoracic pathologic specimen found in this case. These changes can perpetuate respiratory symptoms even in the absence of menstruation [23-25]. Chronic intra-abdominal bleeding from ectopic endometrial tissue can cause macrophage activation and inflammatory cytokine production, resulting in fibrotic adhesions. These dense adhesions may result in strictures or entrapment of pelvic or gastrointestinal organs, causing ileus, obstruction, and infertility [1,25,26]. In severe cases, major intra-abdominal surgery may be required to restore organ function, which ultimately occurred in this case, reflecting the extensive complications of endometriosis when not addressed early on.

## Conclusions

Extra-pelvic endometriosis should be considered in pre-menopausal women presenting with recurrent internal bleeding of unclear etiology, and a thorough menstrual history should be obtained. Delays in diagnosis may lead to chronic inflammatory changes and treatment-refractory symptoms. Early diagnosis and intervention are critical to prevent long-term complications and improve outcomes.
